# Genomic Analysis of Brassinosteroid Biosynthesis Gene Family Reveals Its Roles in Cotton Development across *Gossypium* Species

**DOI:** 10.3390/biology13060380

**Published:** 2024-05-25

**Authors:** Shiyan Cui, Xin Zhou, Guanghui Xiao, Hongjie Feng

**Affiliations:** 1 School of Agricultural Science, Zhengzhou University, Zhengzhou 450001, China; csy9910061999@yeah.net; 2 College of Life Sciences, Shaanxi Normal University, Xi’an 710119, China; zhouxin8852@163.com

**Keywords:** cotton, marker-assisted breeding, fiber development

## Abstract

**Simple Summary:**

Our study systematically investigated the brassinosteroid synthesis enzyme family in four different species of cotton: *Gossypium hirsutum*, *Gossypium barbadense*, *Gossypium arboreum*, and *Gossypium raimondii*, through chromosomal localization, phylogenetic tree construction, promoter motif analysis, protein domain analysis, and evolutionary collinearity analysis. Additionally, we identified and validated potential interacting proteins and their upstream transcription factors, focusing on genes exhibiting high expression levels during fiber development. This comprehensive exploration sheds light on the regulatory role of the brassinosteroid synthesis enzyme family in cotton development, offering molecular insights into its mechanisms.

**Abstract:**

Cotton is a globally significant economic crop. Brassinosteroids (BRs) are crucial to cotton development. This study systematically analyzed the BR synthase gene family in four cotton species and identified 60 BR genes: 20 in *Gossypium hirsutum* (*GhBRs*), 20 in *G. barbadense* (*GbBRs*), 10 in *G. arboreum* (*GaBRs*), and 10 in *G. raimondii* (*GrBRs*). The analysis was extended to chromosomal localization, evolutionary relationships, domain features, and *cis*-regulatory elements in the promoter regions of BR synthase genes. The results showed that the BR synthase genes were evenly distributed across different subgenomes and chromosomes. Bioinformatics analyses revealed high conservation of amino acid sequences, secondary structures, and conserved domains among the subfamily members, which is closely linked to their pivotal roles in the BR biosynthesis pathway. *Cis*-element distribution analysis of the BR synthase genes further underscored the complexity of BR gene expression regulation, which is influenced by multiple factors, including plant hormones, abiotic stress, and transcription factors. Expression profiling of *GhBRs* genes in various cotton tissues and developmental stages highlighted the key roles of *GhROT3-1* and *GhDET2-1* in fiber elongation and initiation, respectively. Protein–protein interactions and transcription factor analyses further elucidated the regulatory mechanisms of GhROT3-1 and GhDET2-1 in cotton growth and development. This study lays a theoretical foundation for understanding the role of the BR signaling pathway in cotton development, facilitating molecular breeding.

## 1. Introduction

Cotton (*Gossypium* spp.), the most significant natural fiber crop globally, is predominantly used in the textile industry [1]. Cotton fibers are extended single cells from the epidermal layer of cotton seeds, and play a crucial role in determining fiber quality, including length and strength [2]. Understanding the complex processes that drive the development of cotton fibers is essential for increasing cotton yields and enhancing fiber quality. Cotton fiber growth has four distinct phases: initiation, elongation, secondary cell wall thickening, and maturation [3]. Throughout these stages, a complex series of biochemical and molecular biological interactions occur, including the synthesis of cell walls, regulation of plant hormones, and control of gene expression [4].

Brassinosteroids (BRs) are crucial steroidal hormones that regulate a wide array of plant developmental and physiological processes [5], including cell elongation, photomorphogenesis, stomatal development, male fertility, and crop yield, as well as resistance to environmental stressors (e.g., drought, salinity, cold, and pathogens). BRs are central to plant growth, and influence cell division and elongation [6]. They shape plant architecture by promoting leaf expansion, stem growth, and flower development. Additionally, BRs play a critical role in plant adaptation to environmental challenges [7]. Research has shown that BRs significantly enhance the elongation of cotton fibers by activating specific signaling pathways and regulating gene expression, improving cell elongation [8]. BRs also strengthen and thicken cotton fibers, improving the structure of the cell walls, and thereby promoting the physical properties and commercial value of the fibers.

The synthesis of brassinosteroids involves a series of enzymatic reactions, which can be broadly categorized into two main pathways: the early C-6 oxidation pathway and the late C-6 oxidation pathway. Biosynthesis of brassinosteroids commences with sterols such as β-sitosterol or campesterol, undergoing cyclization to form campestanol. Subsequently, enzymes such as CYP90B1/DWF4 catalyze the oxidation at the C-6 position, yielding 6-deoxocastasterone (6-DeoxoCS). Further modifications include C-22 hydroxylation catalyzed by CYP90D2 and CYP90A1, as well as C-3 oxidation catalyzed by CYP85A1, ultimately resulting in the formation of castasterone (CS). The final step involves C-6 oxidation catalyzed by CYP85A2, generating biologically active brassinolide (BL). Similarly, the late pathway also initiates with sterols, undergoing cyclization followed by C-22 hydroxylation and C-23 oxidation. These modifications lead to the formation of intermediates such as 6-deoxoteasterone, which undergo further oxidation at the C-6 and C-3 positions catalyzed by enzymes like CYP85A2 and CYP85A1, respectively, ultimately yielding active brassinosteroids [9].

Research has indicated that the biosynthesis of BRs is driven by specific enzymes, such as BR6ox1, BR6ox2, CPD, CYP90D1, DET2, DWF4, and ROT3 [9,10]. The activities of these enzymes are crucial for the efficient production of BRs. In *Arabidopsis thaliana*, the enzymes BR6ox1 and BR6ox2 are primarily involved in the final steps of the BR biosynthetic pathway [10]. Studies on mutants have revealed that deficiencies in these enzymes lead to pronounced dwarfism, delayed growth, small leaves, and abnormal floral organs, highlighting BRs’ critical role in cell expansion and differentiation [11]. For example, the CPD gene encodes a pivotal P450 enzyme central to the BR pathway, with CPD mutants exhibiting severe dwarfism and dark, thick leaves typical of BR deficiency [12]. These mutants also exhibit compromised photomorphogenic responses to light. Similarly, CYP90D1 and DWF4 encode P450 enzymes critical for BR production, and their mutants display growth suppression and severe dwarfism, respectively [13]. DET2 mutants missing an enzyme necessary for an early step in the pathway exhibit extreme dwarfism and delayed flowering. ROT3 affects leaf shape, with ROT3 mutants showing leaves that are narrower and shorter than those typically observed [14]. These variations underscore the essential roles of BRs in plant development and adaptation to stress.

Cotton is an economically vital crop worldwide primarily because of its cotton fibers, which develop via a complex series of biological and molecular activities. BRs are critical hormones for plant growth and play a significant role in this process. Thus, to provide insights into the understanding of the regulatory mechanisms controlling fiber growth, we aimed to investigate the role of the BR synthase enzyme family in the development of cotton fibers [15]. Our research provides a foundation for further studies on the regulatory networks that influence cotton fiber development.

## 2. Materials and Methods

### 2.1. Genome Databases

The *Arabidopsis thaliana* genome sequence used in this study was downloaded from The Arabidopsis Information Resource (TAIR10) database [16]. The genome sequences of *Gossypium hirsutum* (Tx-JGI_G.*hirsutum*_v1.1), *Gossypium raimondii* (D5_NSF), *Gossypium arboreum* (A2_CRI), and *Gossypium barbadense* (3-79_HAU) were obtained from the Cotton Functional Genomics Database (https://yanglab.hzau.edu.cn/CottonMD, accessed on 12 May 2024) [17].

### 2.2. Identification of Brassinosteroid Synthesis Genes in the Cotton Genome

*Arabidopsis thaliana* BR gene IDs were sourced from the literature, and genes were classified into six families—CPD, DWF4, DET2, ROT3, CYP90D1, and BR6ox1/2—according to The Arabidopsis Information Resource (TAIR10). The gene sequences obtained from Arabidopsis were used as bait. Using the TBtools software, these six Arabidopsis families were subjected to local BLASTP analysis against four cotton species (e-value: 1 × 10^−5^; % identity > 55) to obtain cotton IDs and identify putative BR-related homologous gene families [3].

### 2.3. Sequence Analysis of BR Synthesis Genes

To explore the BR domains within four species of cotton, domain sequences were retrieved using the NCBI domain search tool (https://www.ncbi.nlm.nih.gov/Structure/bwrpsb/bwrpsb.cgi, accessed on 12 May 2024). Visualization was performed using the TBtools software. Sequence analysis was conducted with the TBtools software package (Version 2.092) by comparing the candidate genes’ CDS sequences with their genomic DNA sequences. The positions of exons and introns within the genes were extracted from the corresponding GFF files and visualized [17,18].

### 2.4. Phylogenetic Tree Construction of BR Synthesis Genes

To investigate the phylogenetic relationships of BR proteins in the four cotton species, all protein sequences were used to construct a phylogenetic tree using the neighbor-joining method in MEGAX software (Version 7.0.26, http://www.megasoftware.net/mega.php, accessed on 12 May 2024). The Bootstrap value was set at 1000 [18].

### 2.5. Gene Duplication and Homology Analysis

Conserved protein motifs of cotton BR biosynthesis proteins were analyzed using the MEME online tool (http://meme-suite.org/tools/meme, accessed on 12 May 2024). Visualization was carried out with TBtools [19].

### 2.6. Protein Interaction Network Analysis

Protein interaction analysis was conducted using the STRING online tool (https://cn.string-db.org/, accessed on 12 May 2024), and visualizations were performed using Cytoscape software (Version 3.10) [20].

### 2.7. Spatiotemporal Expression Analysis of BRs Synthesis Genes

Expression data for the BR gene family in *Gossypium hirsutum* were retrieved from (https://yanglab.hzau.edu.cn/CottonMD/expression.1, accessed on 12 May 2024).

### 2.8. Statistical Analysis

Averages and standard errors were calculated using Microsoft Excel software (Version 2021). Student’s *t*-tests were performed using SPSS 23.0 to assess the significance of the differences between control and treatment samples or among different time points. A significance threshold was set at *p* < 0.01.

### 2.9. Y1H Assay

In the yeast one-hybrid (Y1H) assay, 2000 bp promoter fragments derived from downstream candidate genes were cloned into the pLacZi vector (Clontech, Mountain View, CA, USA). Coding sequences of candidate transcription factor (TF) genes were inserted into the JGY4-5 vector (Clontech). Both the prey vectors containing the candidate TF gene coding sequences and the bait vectors were co-transformed into the yeast strain EGY48. The transformed yeast cells were evenly plated onto a synthetic dropout medium (Trp/Ura) and cultured on plates supplemented with X-gal for β-galactosidase detection. The cultures were incubated at 30 °C for a period of 3–5 days [21].

### 2.10. In Vivo Dual-LUC Assay

In the dual-luciferase (LUC) reporter assay, promoter fragments spanning 2000 bp upstream of *GhDET2-1* and *GhROT3-1* from *Gossypium hirsutum* genomic DNA were amplified and individually cloned upstream of the firefly luciferase LUC reporter gene in the pGreenII 0800-LUC vector. The coding sequences of candidate transcription factors were cloned as effectors into the pGreenII 62-SK vector. These effector and reporter vectors were transformed into *Agrobacterium tumefaciens* strain GV3101 and transiently co-infiltrated into the epidermal cells of *Nicotiana tabacum* leaves. The activities of LUC and REN (*Renilla luciferase*) were measured using the Dual-Luciferase Reporter Assay System.

## 3. Results

### 3.1. Genome-Wide Identification of BR Gene Family Members in Cotton Species

We identified members of the BR gene family in cotton by using *A. thaliana* BR protein sequences (AtBRs) obtained from The Arabidopsis Information Resource (TAIR; https://www.arabidopsis.org/) database. We compared these sequences against the protein databases of four cotton species: *Gossypium hirsutum* (Tx-JGI_*G.hirsutum*_v1.1), *Gossypium raimondii* (D5_NSF), *Gossypium arboreum* (A2_CRI), and *Gossypium barbadense* (3-79_HAU). This comparison led to the identification of 60 BR genes: 20 in *G. hirsutum*, 20 in *G. barbadense*, 10 in *G. arboreum*, and 10 in *G. raimondii* (Table S1).

### 3.2. Physico-Biochemical Properties Analysis

The physico-chemical analysis of the BR biosynthesis gene family in cotton demonstrated that the amino acid count of the encoded proteins varied within a narrow range. Specifically, the shortest BR protein, GbBR6ox-3, consisted of 224 amino acids, and the longest proteins, GbROT3-3 and GhROT3-3, each had 516 amino acids. The molecular weights of these proteins ranged from 26.47 kDa for GbBR6ox-3 to 58.44 kDa for GbROT3-3 and GhROT3-3. Isoelectric point (*p*I) analysis showed that GbROT3-1 and GrROT3-2 were acidic (*p*I < 7.0) and that the other BR proteins were basic, (*p*I > 7.0). The hydropathy analysis (GRAVY) indicated that GbDET2-1, GaDET2-1, GrDET2-1, GhDET2-1, and GhDET2-2 were hydrophobic, with GRAVY values above zero, and all other BR proteins were hydrophilic, with GRAVY values below zero. Instability index analysis further revealed that all BR proteins had an instability index under 50, 5 members were below 40.0, and 45 members were above 40.0, suggesting that most of them are potentially unstable (Table S2).

### 3.3. Chromosomal Location Analysis of BRs in Cotton Species

**Figure 1:**
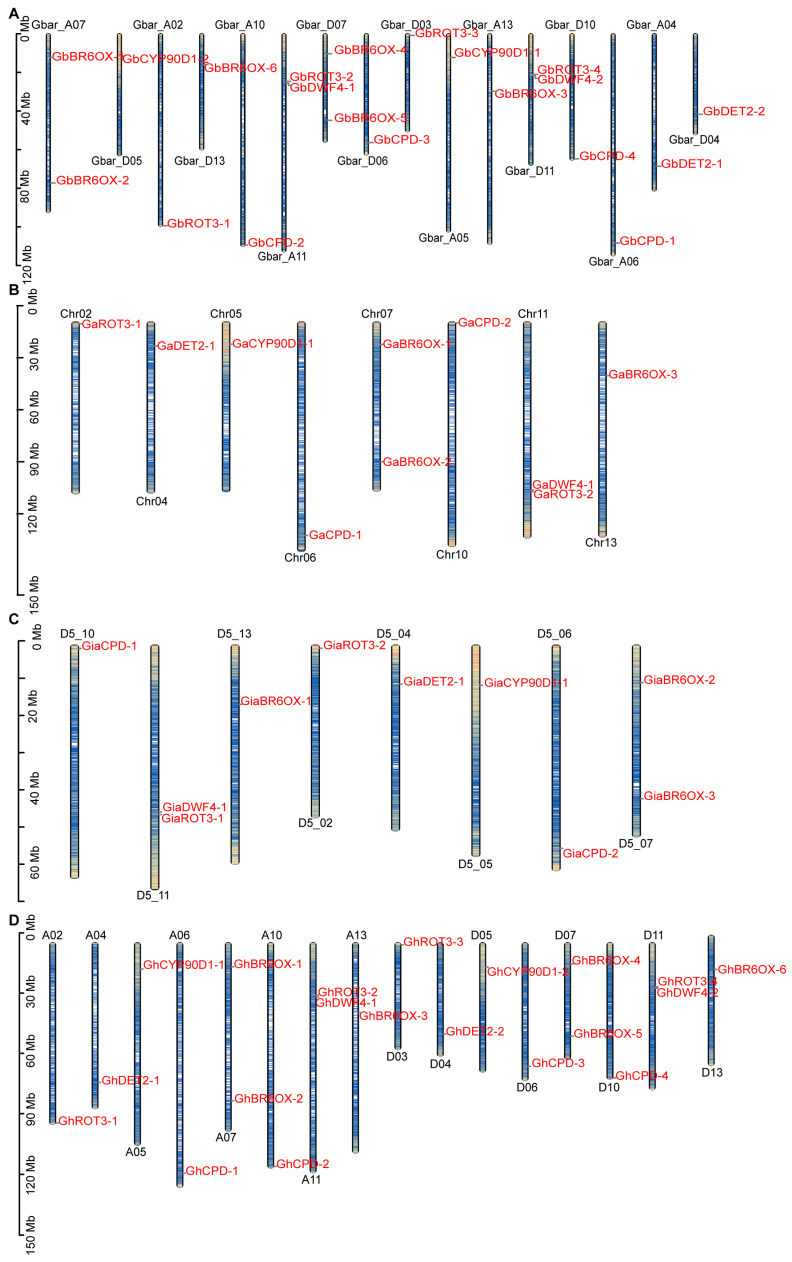
Chromosomal localization analysis of BRs family members: (**A**) Chromosomal localization of BR biosynthesis members from *G. barbadense* (Gb). (**B**) Chromosomal localization of BR biosynthesis members from *G. arboretum* (Ga). (**C**) Chromosomal localization of BR biosynthesis members from *G. raimondii* (Gr). (**D**) Chromosomal localization of BR biosynthesis members from *G. hirsutum* (Gh). Mb, Mega base pair.

Utilizing GFF files from *G. hirsutum* (Tx-JGI_G.*hirsutum*_v1.1), *G. raimondii* (D5_NSF), *G. arboreum* (A2_CRI), and *G. barbadense* (3-79_HAU), we mapped the chromosomal locations of genes by using the MG2C online tool (Figure 1). The analysis revealed that 20 genes from *GbBRs* were uniformly distributed across 16 chromosomes, with half located in subgenome A and half in subgenome D (Figure 1A). The *GaBRs*, comprising 10 genes, were also evenly spread across 8 chromosomes, typically exhibiting from 1 to 2 genes per chromosome, except for chromosomes Chr07 and Chr11, which each contained 2 genes (Figure 1B). A similar pattern was observed for *GrBRs*, in which 10 genes were spread across 8 chromosomes (Figure 1C). *GhBRs* had the same distribution as *GbBRs*, with 20 genes evenly distributed across 16 chromosomes (Figure 1D). These patterns suggest gene deletions and duplications within the BR gene family across cotton species.

### 3.4. Phylogenetic Analysis of BRs

**Figure 2:**
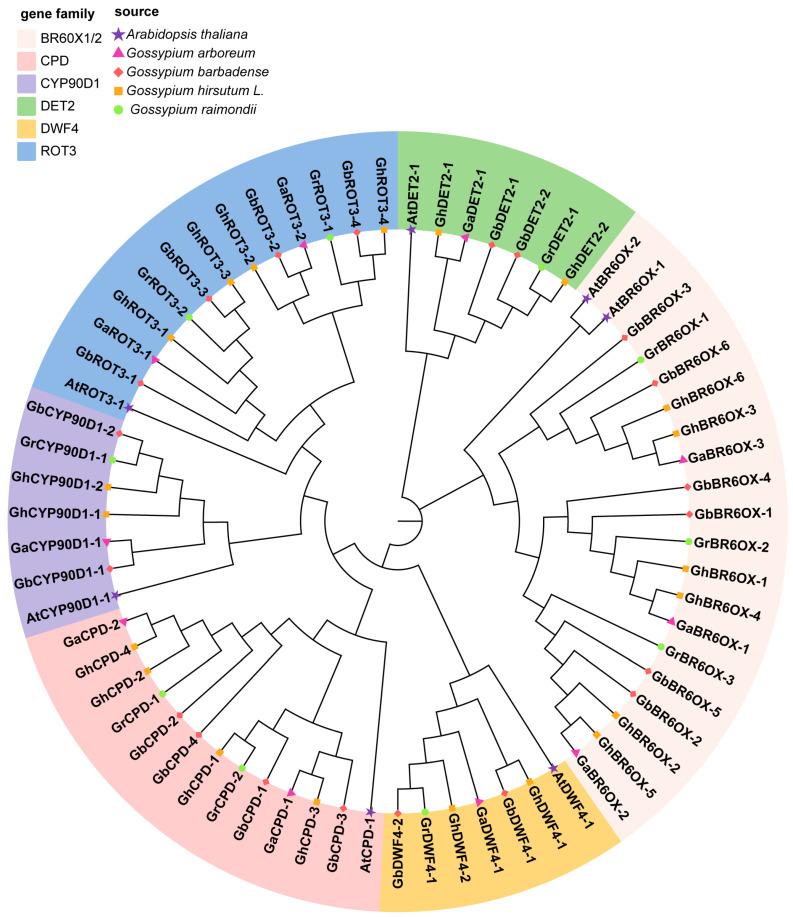
Phylogenetic tree of BRs family members from Arabidopsis and four cotton species. The protein sequences were selected from *G. hirsutum* (Gh), *G. arboretum* (Ga), *G. barbadense* (Gb), *G. raimondii* (Gr), and *A. thaliana* (At). The phylogenetic tree was constructed by MEGA 7.0 software using the Neighbor-Joining (NJ) method with bootstrap replicates set to 1000.

To explore the evolutionary relationships among BRs, we uploaded the full-length protein sequences of 60 BRs, including those from *A. thaliana*, into MEGA7.0. We aligned these sequences and constructed a phylogenetic tree by using the neighbor-joining method. This tree helped classify the BR protein sequences from *G. hirsutum*, *G. arboreum*, *G. raimondii*, *G. barbadense*, and *A. thaliana* into various subgroups (Figure 2). Phylogenetic analysis showed that the BR proteins from these five species were divided into six subfamilies: BR6ox1/2, CPD, CYP90D1, DET2, DWF4, and ROT3. The DWF4, CYP90D1, and DET2 subfamilies had the smallest membership, each having 7 members; the CPD and ROT3 subfamilies each had 13 members; and the BR6ox1/2 subfamily included the largest group, with 20 members.

### 3.5. Protein Features of BRs

**Figure 3:**
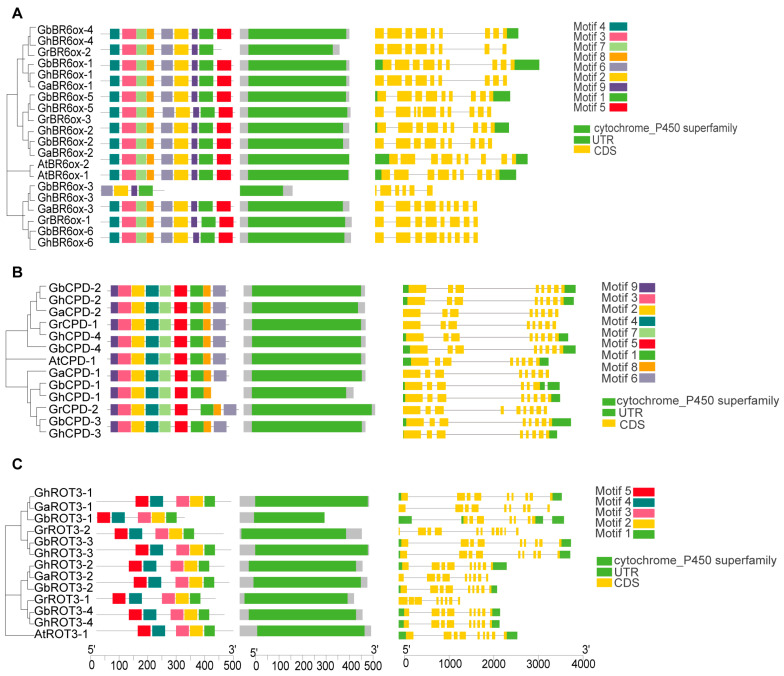
Sequence features of BRs members: (**A**) The conserved motifs distribution and exon–intron distribution of BR6ox1/2 protein sequences. (**B**) The conserved motif distribution and exon–intron distribution of CPD protein sequences. (**C**) The conserved motif distribution and exon–intron distribution of ROT3 protein sequences.

**Figure 4:**
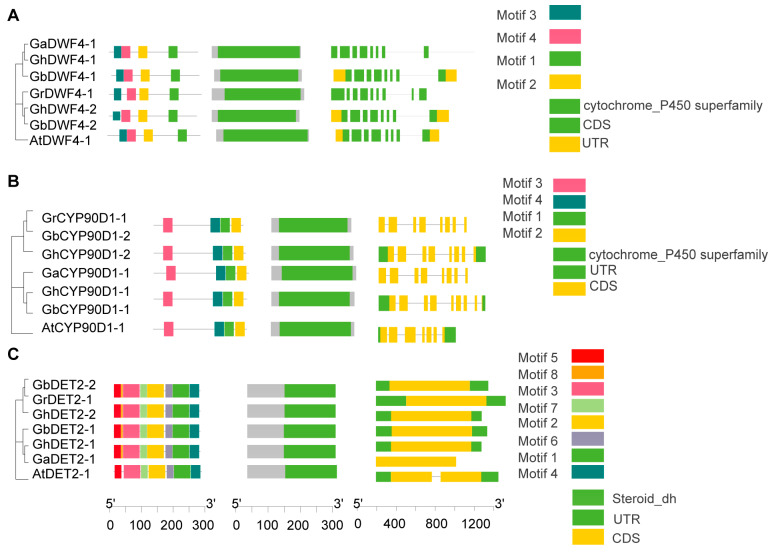
Sequence features of BRs members: (**A**) The conserved motif distribution and exon–intron distribution of DWF4 protein sequences (**B**) The conserved motif distribution and exon–intron distribution of CYP90D1 protein sequences. (**C**) The conserved motif distribution and exon–intron distribution of DET2 protein sequences.

To analyze the conserved structural domains within BRs, we categorized 60 BR proteins into six subfamilies—BR6ox1/2, CPD, CYP90D1, DET2, DWF4, and ROT3—and analyzed their sequences using the NCBI’s Conserved Domain Database (CDD). Each subfamily had a distinct conserved domain. Specifically, the BR6ox1/2 subfamily featured a cytochrome P450 monooxygenase domain crucial for catalyzing key oxidation reactions in BR biosynthesis (Figure 3A). The CPD subfamily included an FAD/NAD(P)-binding domain, essential for dehydrogenation processes in BR synthesis (Figure 3B). The ROT3 subfamily also had a cytochrome P450 domain, which supports hydroxylation in the BR pathway (Figure 3C). Similarly, the DWF4 subfamily possessed cytochrome P450 monooxygenase domains aligned with their roles in BR biosynthesis (Figure 4A). The CYP90D1 subfamily contains a similar cytochrome P450 domain that facilitates hydroxylation (Figure 4B), and the DET2 subfamily primarily includes a short-chain dehydrogenase domain for reductive functions in the pathway (Figure 4C). These analyses highlight extensive structural conservation across BR gene subfamilies, emphasizing their critical functions in BR biosynthesis.

### 3.6. Cis-Promoter Element Analysis of BRs

**Figure 5:**
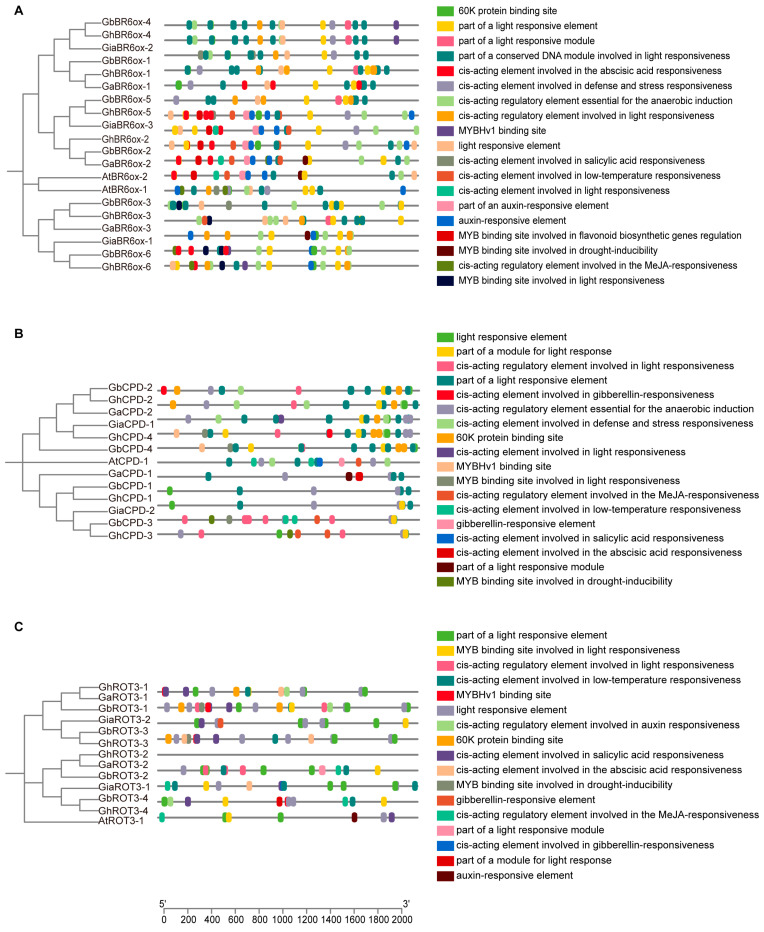
Cis-promoter element analysis of BR6ox1/2, CPD, and ROT3: (**A**) Cis-promoter element analysis of BR6ox1/2 sequences from cotton and Arabidopsis. (**B**) Cis-promoter element analysis of CPD sequences from cotton and Arabidopsis. (**C**) Cis-promoter element analysis of ROT3 sequences from cotton and Arabidopsis.

**Figure 6:**
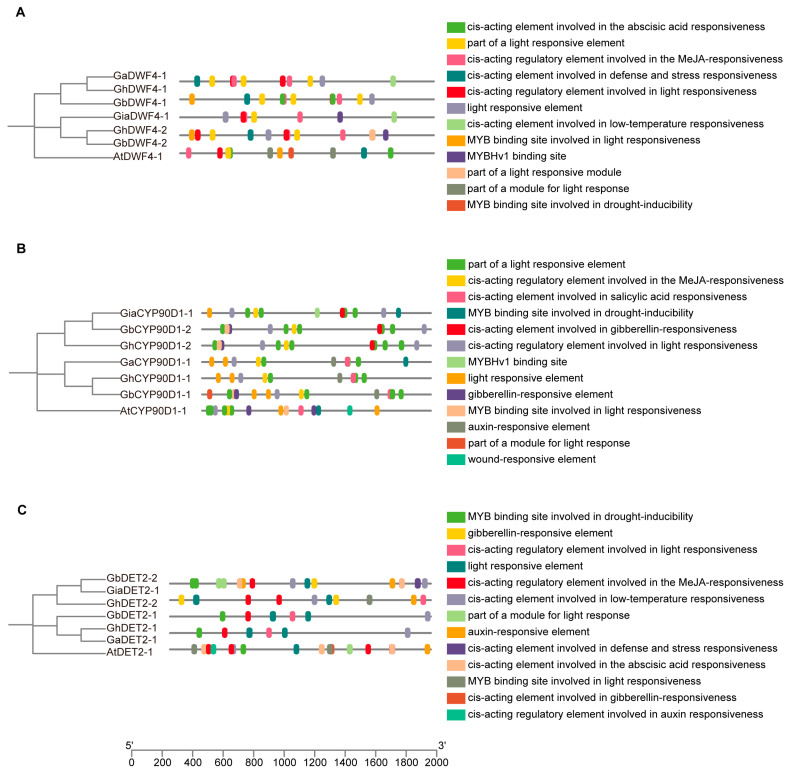
Cis-promoter element analysis of DWF4, CYP90D1, and DET2: (**A**) Cis-promoter element analysis of DWF4 sequences from cotton and Arabidopsis. (**B**) Cis-promoter element analysis of CYP90D1 sequences from cotton and Arabidopsis. (**C**) Cis-promoter element analysis of DET2 sequences from cotton and Arabidopsis.

We analyzed the cis-regulatory elements located 2000 bp upstream of the BRs genes across the four cotton species (Figure 5 and Figure 6). The promoters of these genes were rich in hormone-responsive elements, with gibberellin, auxin, salicylic acid, and abscisic acid being particularly prevalent. Additionally, many elements were related to abiotic stress, including response to low temperatures, anaerobic conditions, and wounding.

The promoter regions also featured binding sites for various transcription factors, such as the 60 K protein, Myeloblastosis (MYB), and AT-rich DNA binding protein (ATBP), indicating a complex regulatory network. Notably, light-responsive elements were present, underscoring the influence of environmental factors on BR gene regulation. This intricate architecture highlights the crucial roles of BRs in plant growth, development, and adaptation to environmental changes.

The co-occurrence of elements responsive to gibberellins and auxins suggests potential synergistic or antagonistic interactions that may affect cell elongation and differentiation. The presence of abiotic stress-responsive elements highlights the multifunctional role of BR genes in response to environmental challenges, such as cold and flooding.

Moreover, the diversity of transcription factor binding sites underlined the complexity of gene expression regulation in BRs. These sites suggest interactions among various transcription factors that can integrate multiple signaling pathways within plants. Additionally, light-responsive elements linked BR gene expression to environmental light conditions, providing a new avenue for exploring how light signals and plant hormones interact to optimize plant growth strategies under variable light scenarios.

Further research should focus on elucidating the changes in the activity of these cis-acting elements across growth stages and environmental conditions and define their specific functions and regulatory mechanisms within the plant’s life processes.

### 3.7. Collinearity Analysis of BRs

**Figure 7:**
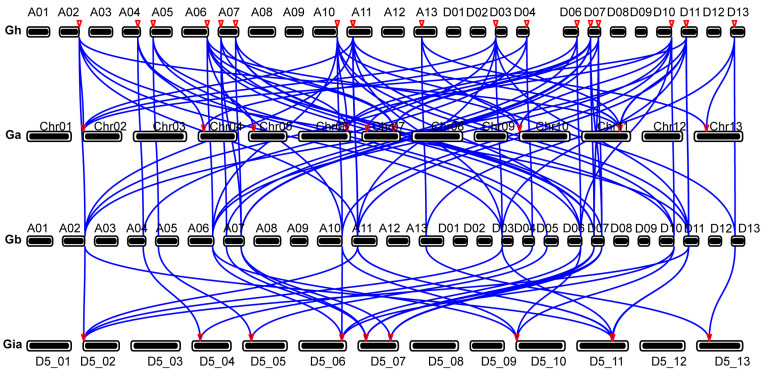
Collinearity of BRs biosynthesis genes in *G. arboretum* (Ga), *G. hirsutum* (Gh), and *G. raimondii* (Gr). The syntenic relationship of different BR biosynthesis genes is connected by blue-colored lines.

Tandem and segmental DNA replications are crucial drivers of gene family expansion and significantly influence genomic evolution. To explore how these mechanisms have affected the evolution of diploid and tetraploid cotton BRs, we conducted a synteny analysis of these cotton types. A total of 9 BR genes from the diploid *G. arboreum* were syntenic with 17 BR genes from the tetraploid *G. hirsutum*, and another set of 18 BR genes from *G. hirsutum* corresponded to 9 BR genes from the diploid *G. raimondii* (Figure 7). These results further suggest that each BR gene in the diploid species has at least one ortholog in the At and Dt subgenomes of the corresponding tetraploid species, highlighting the role of gene duplication and chromosomal rearrangements in the expansion and evolution of the gene family during cotton polyploidization.

### 3.8. Expression Analysis of GhBRs Genes during Cotton Development

**Figure 8:**
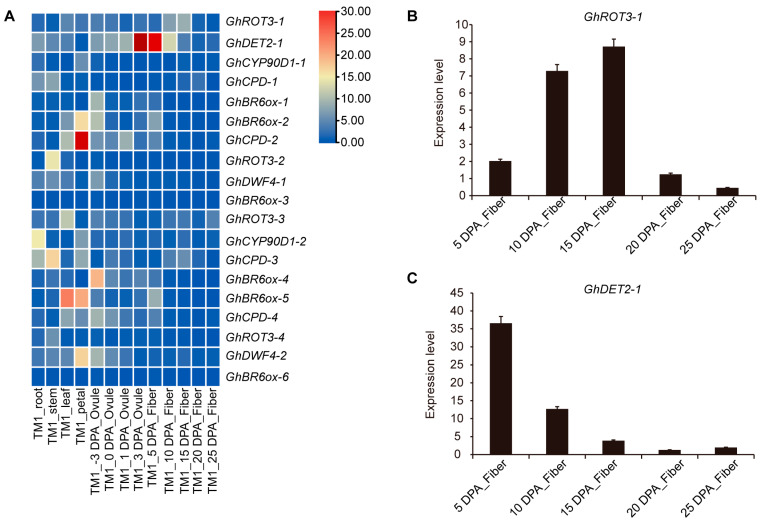
Expression profiles of GhBR biosynthesis family members in different tissues and in fibers at different development stages: (**A**) Expression heatmap of cotton BR biosynthesis members in different tissues and in fibers at different development stages. (**B**,**C**) Relative expression levels of GhROT3-1 and GhDET2 candidate genes in fibers at different development stages. Error bar represents ± SD (*n* = 3). dpa, day post-anthesis.

Upland cotton (*G. hirsutum*) is a major global economic crop that dominates the market for cotton and natural short staple fibers. This study aimed to understand the roles of specific *GhBRs* gene family members across cotton tissues. We analyzed the expression profiles of these genes in the roots, stems, leaves, flowers, ovules, and at various stages of fiber development (Figure 8A). *GhROT3-1* was predominantly expressed in 15-day-old fibers, suggesting a role in fiber elongation. *GhDET2-1* was mainly expressed in 5-day-old fibers, implying its involvement in fiber initiation. *GhBR6ox-2*, *GhCPD-2*, *GhBR6ox-5*, and *GhDWF4-2* were significantly expressed in flowers. *GhBR6ox-5* was highly expressed in leaves, and *GhCPD-3* and *GhROT3-2* were highly expressed in stems. Additionally, *GhCPD-3* and *GhCYP90D1-2* were notably expressed in the roots, highlighting the diverse roles that these genes play in the growth and development of upland cotton.

Additionally, we conducted targeted analyses of the expression patterns of *GhROT3-1* and *GhDET2-1* at various stages of fiber development using quantitative real-time PCR (Figure 8B,C). *GhROT3-1* was predominantly expressed during the elongation phase of the fiber, and *GhDET2-1* exhibited increased expression during the initiation phase. These observations provide a foundation for further investigations into their respective roles in the development of cotton fibers.

### 3.9. Prediction Analysis of GhROT3-1 and GhDET2-1 Protein–Protein Interaction (PPI)

**Figure 9:**
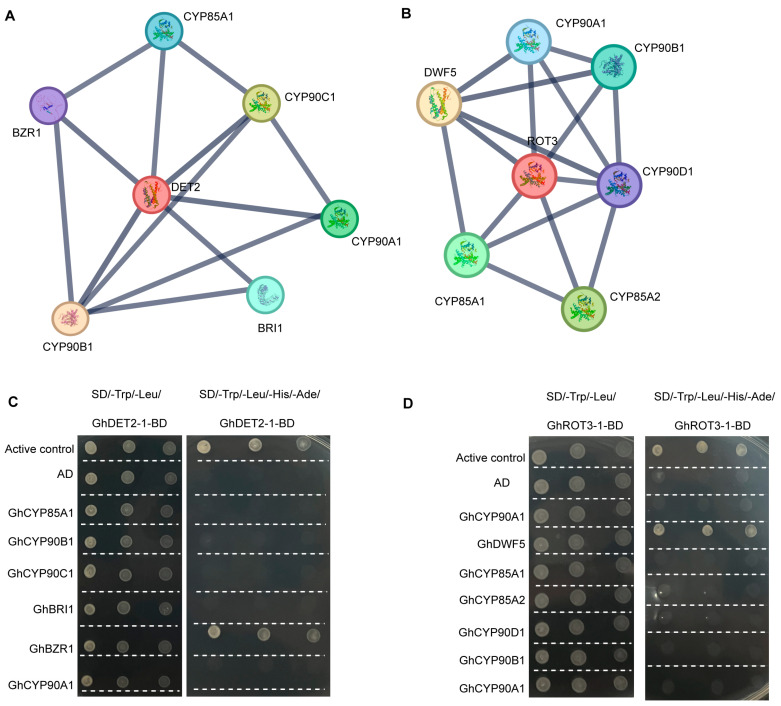
Protein–protein interaction (PPI) network and yeast two-hybrid verification experiment of BR biosynthesis members: (**A**) Protein–protein interaction network of DET2 in cotton. (**B**) Protein–protein interaction network of ROT3 in cotton. (**C**) Yeast two-hybrid showing the interaction of DET2 and six candidate genes. (**D**) Yeast two-hybrid showing the interaction of ROT3 and seven candidate genes PPI networks were analyzed using the STRING database 10.5.

To elucidate the functions of *GhROT3-1* and *GhDET2-1* in *G. hirsutum*, we constructed a PPI network of these two genes. The analysis revealed that GhDET2-1 may interact with BZR1, CYP85A1, BRI1, CYP90A1, CYP90C1, and CYP90B1 (Figure 9A), and GhROT3-1 may interact with CYP90D1, CYP90A1, CYP90B1, CYP85A1, CYP85A2, and DWF5 (Figure 9B). To validate these potential interactions, The results demonstrated a clear interaction between GhDET2-1 and BZR1 (Figure 9C), and a significant interaction between GhROT3-1 and DWF5 (Figure 9D). These findings not only enhance the understanding of the key gene interaction networks in upland cotton but also provide important molecular insights for further functional studies and breeding applications.

### 3.10. Prediction and Validation of Transcription Factors Regulating Genes GhROT3-1 and GhDET2-1

**Figure 10:**
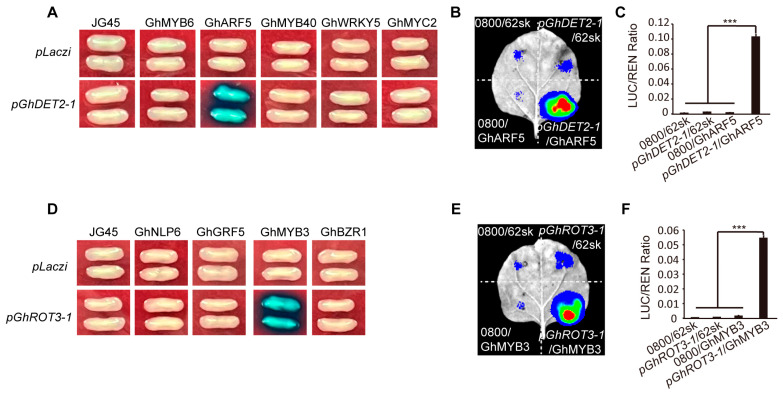
Prediction and validation of transcription factors regulating the genes GhROT3-1 and GhDET2-1: (**A**) Yeast one-hybrid assays of GhDET2-1 promoter and five transcription factors. (**B**) Transient expression assay in *N. benthamiana* leaves showing the transcriptional activation of the LUC reporter gene (driven by the GhDET2-1 promoter) by the GhARF5. (**C**) Quantification of LUC activity in (**B**). Renilla (REN) activity was used for normalization. The LUC/REN ratio indicates the relative activity of the promoter. (**D**) Yeast one-hybrid assays of GhROT3-1 promoter and five transcription factors. (**E**) Transient expression assay in *N. benthamiana* leaves showing the transcriptional activation of the LUC reporter gene (driven by the GhROT3-1 promoter) by the GhMYB3. (**F**) Quantification of LUC activity in (**E**). Renilla (REN) activity was used for normalization. The LUC/REN ratio indicates the relative activity of the promoter. Statistical significance for each comparison is indicated (*t*-test): ***, *p* ≤ 0.001.

We utilized the PlantCARE resource (https://bioinformatics.psb.ugent.be/webtools/plantcare/html/, accessed on 13 May 2024) to predict the upstream transcription factors of *GhROT3-1* and *GhDET2-1*, and identified 10 potential target genes. To validate these findings, we performed yeast one-hybrid experiments of the 2000 bp promoter regions upstream of the target genes and the coding sequences of the candidate genes. The experiments showed that GhMYB3 interacted with the promoter region of *GhROT3-1* (Figure 10A), and GhARF5 interacted with the promoter sequence of *GhDET2-1* (Figure 10D). These interactions were confirmed by dual-luciferase assays, which provided additional validation of the synergistic effects between these genes by measuring the luciferase (LUC) activity in leaf tissues (Figure 10B,C,E,F).

## 4. Discussion

This study provides comprehensive insights into the BR gene family in four major cotton species: *G. hirsutum*, *G. barbadense*, *G. arboreum*, and *G. raimondii*. This study also significantly expands the understanding of BR biosynthesis genes in cotton, contributing to the literature on this important plant hormone family.

Our analysis of the cotton BR gene family revealed several notable differences and similarities between our findings and those in the literature on model plants, such as Arabidopsis. For instance, we identified 60 BR genes in the four cotton species examined, and the literature on model plants, such as Arabidopsis, has reported 11 BR biosynthesis genes [22]. This disparity highlights the expansive nature of the BR gene family in the cotton genome, probably driven by gene duplication and diversification events during the evolutionary history of cotton.

Notably, the distribution and chromosomal localization patterns of BR genes in cotton resemble those reported in rice, another major crop plant [23]. The study demonstrated an even distribution of BR genes across chromosomes, suggesting conserved mechanisms for gene family expansion and genome organization during the evolution of monocots and dicots.

The domain architecture analysis performed in this study aligns well with the characterizations of BR biosynthesis enzymes in the literature. The identified conserved domains, such as the cytochrome P450 monooxygenase and short-chain dehydrogenase domains, are consistent with the known catalytic roles of these proteins in key BR biosynthetic steps [24]. This structural conservation across BR subfamilies underscores the fundamental importance of these domains in maintaining the integrity of the BR biosynthetic pathway.

Expression profiling of *GhBR* genes revealed distinct temporal and spatial patterns, highlighting their diverse roles in cotton development. Notably, the identified roles of *GhROT3-1* and *GhDET2-1* in fiber elongation and initiation, respectively, provide valuable insights into the regulation of this economically important trait. These findings corroborate those of studies on Arabidopsis and other plant species, in which BR biosynthesis genes influence various growth and developmental processes [25].

In this study, PPI analysis and transcription factor validation experiments further elucidated the complex regulatory networks governing BR biosynthesis in cotton. The identified interactions between GhDET2-1 and BZR1, GhROT3-1 and DWF5, aligned with known BR signaling components in model plants. Similarly, the transcriptional regulation of *GhROT3-1* by GhMYB3 and *GhDET2-1* by GhARF5 provides novel insights into the upstream regulation of BR biosynthesis genes in cotton.

This study provides a more comprehensive and integrated understanding of the BR gene family in cotton than that from the literature on BR biosynthesis in other plant species. The combination of genome-wide identification, structural and evolutionary analyses, expression profiling, and regulatory network characterization provides a robust framework for understanding the multifaceted roles of BRs in cotton growth, development, and fiber production.

Further research should focus on validating the functional roles of specific BR biosynthesis genes through reverse genetics approaches such as gene knockout or overexpression studies. Additionally, investigating the dynamic expression patterns of BR genes under various environmental conditions and stress treatments may provide insights into their adaptive functions in cotton. Elucidating the intricate signaling crosstalk between BRs and other phytohormones, as well as the transcriptional regulatory mechanisms governing BR biosynthesis, would further enhance the understanding of this important hormone family in economically valuable crops.

## 5. Conclusions

In this study, we conducted a bioinformatics analysis that identified 60 BR genes across four species of cotton, demonstrating the widespread distribution and conservation of the BR gene family within the genus Gossypium. Further analysis of physico-chemical properties indicated that the proteins encoded by these BR genes possess a relatively narrow range of amino acid counts, molecular weights, and isoelectric points.

Chromosomal localization analysis revealed a uniform distribution of the BR gene family members across the chromosomes of cotton, suggesting that gene duplication and deletion events may have driven the expansion of the BR gene family during cotton evolution. Phylogenetic analysis divided the BR genes into six subfamilies, revealing the differentiation and conservation of this gene family throughout evolutionary processes.

Cis-regulatory element analysis indicated that the expression of BR genes is subject to complex regulation by various plant hormones and environmental factors, which play crucial roles in processes such as plant growth, development, and stress responses. This result provides important clues for future exploration of the molecular mechanisms that regulate BR gene functions.

Collinearity analysis suggested that gene duplications and chromosomal rearrangements were the drivers of the expansion of the BR gene family during cotton polyploidization. Expression analysis further elucidated the functional differentiation of the BR gene family members in various tissues and developmental stages of cotton, with high expression of GhROT3-1 during the fiber elongation phase and GhDET2-1 during the early stages of fiber development.

Protein interaction network predictions and validations showed interactions between GhROT3-1 and DWF5 and between GhDET2-1 and BZR1, revealing the molecular mechanisms by which these key BR genes regulate cotton development. Transcription factor prediction and validation further confirmed that GhMYB3 and ARF5 regulate GhROT3-1 and GhDET2-1 expression, respectively.

This comprehensive study elucidated the genome, evolutionary characteristics, regulation of expression, and functional interaction networks of the cotton BR gene family. This research lays a solid foundation to deepen the understanding of the role of the BR signaling pathway in cotton growth and development and provides crucial references for future functional validation and molecular breeding applications.

## Data Availability

Data available in a publicly accessible repository.

## Supplementary Materials

The following supporting information can be downloaded at: https://www.mdpi.com/article/10.3390/biology13060380/s1, Table S1: The id of BRs; Table S2: The information of BRs.

## References

[1] Jan M., Liu Z., Guo C., Sun X. (2022). Molecular Regulation of Cotton Fiber Development: A Review. Int. J. Mol. Sci..

[2] Ruan Y.L. (2013). Boosting seed development as a new strategy to increase cotton fiber yield and quality. J. Integr. Plant Biol..

[3] Wang M., Sun R., Li C., Wang Q., Zhang B. (2017). MicroRNA expression profiles during cotton (*Gossypium hirsutum* L.) fiber early development. Sci. Rep..

[4] Yang Z., Liu Z., Ge X., Lu L., Qin W., Qanmber G., Li F. (2023). Brassinosteroids regulate cotton fiber elongation by modulating very-long-chain fatty acid biosynthesis. Plant Cell.

[5] Shi Z., Chen X., Xue H., Jia T., Meng F., Liu Y., Zhu S. (2022). GhBZR3 suppresses cotton fiber elongation by inhibiting very-long-chain fatty acid biosynthesis. Plant J..

[6] Lu R., Zhang J., Liu D., Wei Y.L., Wang Y., Li X.B. (2018). Characterization of bHLH/HLH genes that are involved in brassinosteroid (BR) signaling in fiber development of cotton (*Gossypium hirsutum*). BMC Plant Biol..

[7] Sun Y., Veerabomma S., Abdel-Mageed H.A., Fokar M., Asami T., Yoshida S., Allen R.D. (2005). Brassinosteroid regulates fiber development on cultured cotton ovules. Plant Cell Physiol..

[8] Luo M., Xiao Y., Li X., Lu X., Deng W., Li D., Pei Y. (2007). GhDET2, a steroid 5alpha-reductase, plays an important role in cotton fiber cell initiation and elongation. Plant J..

[9] Peres A.L.G.L., Soares J.S., Tavares R.G., Righetto G., Zullo M.A.T., Mandava N.B., Menossi M. (2019). Brassinosteroids, the Sixth Class of Phytohormones: A Molecular View from the Discovery to Hormonal Interactions in Plant Development and Stress Adaptation. Int. J. Mol. Sci..

[10] Zheng L., Zhao C., Mao J., Song C., Ma J., Zhang D., Han M., An N. (2018). Genome-wide identification and expression analysis of brassinosteroid biosynthesis and metabolism genes regulating apple tree shoot and lateral root growth. J. Plant Physiol..

[11] Hamasaki H., Ayano M., Nakamura A., Fujioka S., Asami T., Takatsuto S., Shimada Y. (2020). Light Activates Brassinosteroid Biosynthesis to Promote Hook Opening and Petiole Development in *Arabidopsis thaliana*. Plant Cell Physiol..

[12] Zhang C., He M., Wang S., Chu L., Wang C., Yang N., Xu F. (2021). Boron deficiency-induced root growth inhibition is mediated by brassinosteroid signalling regulation in Arabidopsis. Plant J..

[13] Asami T., Nakano T., Fujioka S. (2005). Plant brassinosteroid hormones. Vitam. Horm..

[14] Ohnishi T., Godza B., Watanabe B., Fujioka S., Hategan L., Ide K., Mizutani M. (2012). CYP90A1/CPD, a brassinosteroid biosynthetic cytochrome P450 of Arabidopsis, catalyzes C-3 oxidation. J. Biol. Chem..

[15] Polko J.K., Pierik R., van Zanten M., Tarkowská D., Strnad M., Voesenek L.A., Peeters A.J. (2013). Ethylene promotes hyponastic growth through interaction with ROTUNDIFOLIA3/CYP90C1 in Arabidopsis. J. Exp. Bot..

[16] Zhu L., Wang H., Zhu J., Wang X., Jiang B., Hou L., Xiao G. (2023). A conserved brassinosteroid-mediated BES1-CERP-EXPA3 signaling cascade controls plant cell elongation. Cell Rep..

[17] Yang Z., Wang J., Huang Y., Wang S., Wei L., Liu D., Weng Y., Xiang J., Zhu Q., Yang Z. (2023). CottonMD: A multi-omics database for cotton biological study. Nucleic Acids Res..

[18] Lamesch P., Berardini T.Z., Li D., Swarbreck D., Wilks C., Sasidharan R. (2012). The Arabidopsis Information Resource (TAIR): Improved gene annotation and new tools. Nucleic Acids Res..

[19] Chen C., Wu Y., Li J., Wang X., Zeng Z., Xu J., Xia R. (2023). TBtools-II: A “one for all, all for one” bioinformatics platform for biological big-data mining. Mol. Plant.

[20] Kumar S., Stecher G., Li M., Knyaz C., Tamura K. (2018). MEGA X: Molecular Evolutionary Genetics Analysis across Computing Platforms. Mol. Biol. Evol..

[21] Price J.L., Ziv O., Pinckert M.L., Lim A., Miska E.A. (2024). rnaCrosslinkOO: An Object-Oriented R Package for the Analysis of RNA Structural Data Generated by RNA Crosslinking Experiments. Bioinformatics.

[22] Shannon P., Markiel A., Ozier O., Baliga N.S., Wang J.T., Ramage D., Ideker T. (2003). Cytoscape: A software environment for integrated models of biomolecular interaction networks. Genome Res..

[23] Ji X., Wang L., Zang D., Wang Y. (2018). Transcription Factor-Centered Yeast One-Hybrid Assay. Methods Mol. Biol..

[24] Choe S., Dilkes B.P., Fujioka S., Takatsuto S., Sakurai A., Feldmann K.A. (1998). The DWF4 gene of Arabidopsis encodes a cytochrome P450 that mediates multiple 22alpha-hydroxylation steps in brassinosteroid biosynthesis. Plant Cell.

[25] Kim T.W., Hwang J.Y., Kim Y.S., Joo S.H., Chang S.C., Lee J.S., Takatsuto S., Kim S.K. (2005). Arabidopsis CYP85A2, a cytochrome P450, mediates the Baeyer-Villiger oxidation of castasterone to brassinolide in brassinosteroid biosynthesis. Plant Cell.

